# Internal Hernia as a Cause of Acute Abdomen in a Pediatric Patient

**DOI:** 10.7759/cureus.14799

**Published:** 2021-05-02

**Authors:** Vignesh Sankar, Aiman Sajjad, Francis Amador

**Affiliations:** 1 Pediatric Surgery, Broward Health Medical Center/Nova Southeastern University Kiran C. Patel College of Osteopathic Medicine, Fort Lauderdale, USA; 2 Medicine, Broward Health Medical Center/Nova Southeastern University Kiran C. Patel College of Osteopathic Medicine, Fort Lauderdale, USA; 3 Pediatric Emergency Medicine, Broward Health Medical Center, Fort Lauderdale, USA

**Keywords:** pediatric laparoscopic surgery, mesenteric internal hernia, small bowel obstruction, small bowel ischemia, coffee-ground emesis, pediatric emergency department, small bowel resection, short bowel, strangulated internal hernia, appendicitis

## Abstract

An acute abdomen is a complex case with multiple possible etiologies and requires the help of many different disciplines. We present the case of a two-year-old female who presented to the emergency department in acute distress, pale in complexion, and continuously guarding her abdomen. Physical examination revealed a distended, rigid abdomen with tenderness to palpation of the abdomen in all four quadrants. A computed tomography scan illustrated markedly dilated loops of small bowel but unclear etiology of obstruction with no evidence of perforation. Stat diagnostic laparotomy showed a strangulated internal hernia secondary to a congenital mesenteric defect. The mesenteric defect was repaired laparoscopically, and 25 cm of necrotic bowel was resected with an end-to-end anastomosis. Internal hernias secondary to mesenteric defects are the most common forms of internal hernias in pediatric patients and present with a 100% mortality rate if left untreated. This case illustrates the importance of a high index of suspicion, thorough physical examination, prompt diagnosis, and treatment in preventing a fatal outcome in these patients.

## Introduction

An acute abdomen is a condition requiring prompt intervention. The etiology of this condition varies from infection to vascular occlusion, to obstruction and is often a clinical diagnosis with most of these patients appearing extremely ill on physical examination. The most common etiology of an acute abdomen in the pediatric population is appendicitis; however, the patient we are presenting in this case report developed an acute abdomen due to strangulation of an internal hernia due to a congenital absence of mesentery. Martin et al. found that these transmesenteric hernias are the most common presenting internal hernias with a 35% incidence rate, 5% risk of incarceration, and greater than 50% overall mortality [1]. These cases require prompt surgical intervention often including necrotic bowel resection, re-anastomosis, and closure of mesenteric defects to prevent a recurrence. These patients are also placed on stringent courses of intravenous antibiotics but do not need to be routinely evaluated after discharge due to the low rate of recurrence.

## Case presentation

We present a two-year-old female with no past medical history who was transferred from an outside facility after 15 episodes of projectile emesis which progressed from a clear to a coffee-ground color. According to the mother, the patient was afebrile at home with no sick contacts, no rash, no recent travel, no pets in the house, no diarrhea, or constipation. However, she complained of nausea and her mother noted significant fatigue and lethargy. Her last bowel movement was two nights prior to arrival. The patient was unable to articulate her own symptoms due to diffuse and sharp pain located in her abdomen. She lived at home with her mom, dad, and three dogs. She had no known drug allergies.

On examination, the patient was visibly in acute distress. She was pale, uncomfortable, and restless in bed with continual abdominal guarding. On palpation, her abdomen was hard, distended, and diffusely tender with severity being highest in the right lower quadrant. Her vitals revealed sinus tachycardia at a regular rate of 176 beats per minute, blood pressure 96/71 mmHg, respiratory rate 25 breaths per minute, temperature at 100.6°F (orally), and oxygen saturation at 98% on room air.

**Figure 1:**
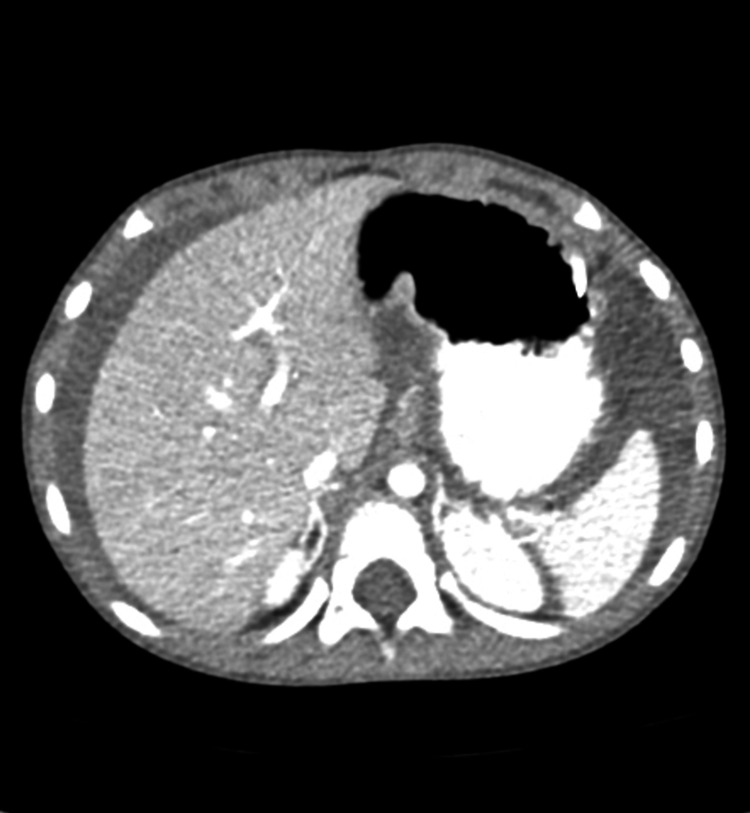
CT scan of the abdomen and pelvis showing a markedly dilated area of small bowel proximal to the obstructed area. CT: computed tomography

**Figure 2:**
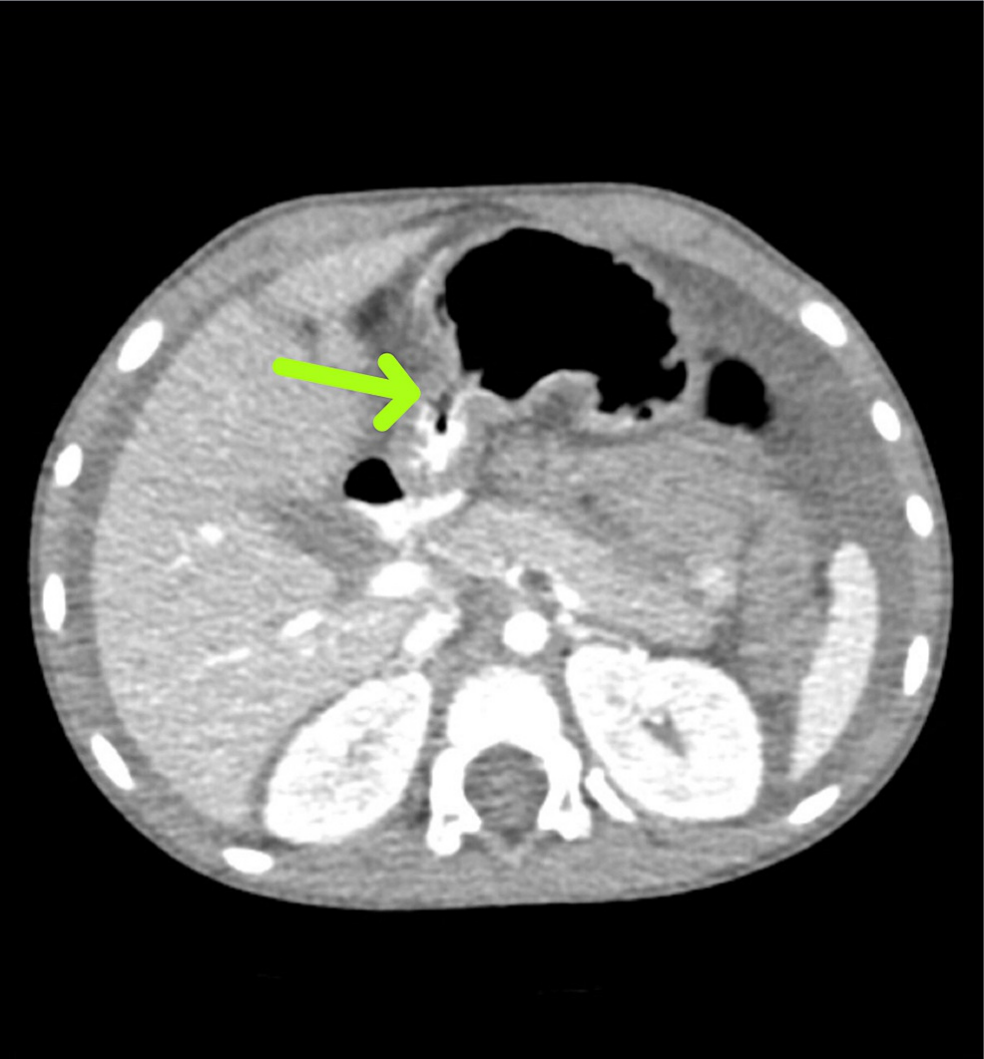
CT scan of the abdomen and pelvis showing possible transition point of obstruction distal to the dilated region of small bowel. CT: computed tomography

The patient’s laboratory studies were remarkable for a white blood cell count of 11.62 × 10^9^ cells/L, sodium at 133 meq/L, potassium at 5.4 meq/L, and bicarbonate at 16 mmol/L with an anion gap of 10 meq/L. Ultrasound of the abdomen showed free fluid throughout the abdomen with several thickened bowel wall loops, no evidence of intussusception, and no visualization of the appendix. The patient was then rushed for a computed tomography (CT) of the abdomen without contrast which showed abnormal dilatation of mid-to-distal small bowel loops with no clear point of transition (Figures 1, 2) suggesting a possible small bowel obstruction or perforated appendicitis; however, the appendix was still unable to be visualized. There was no evidence of bowel perforation.

**Figure 3:**
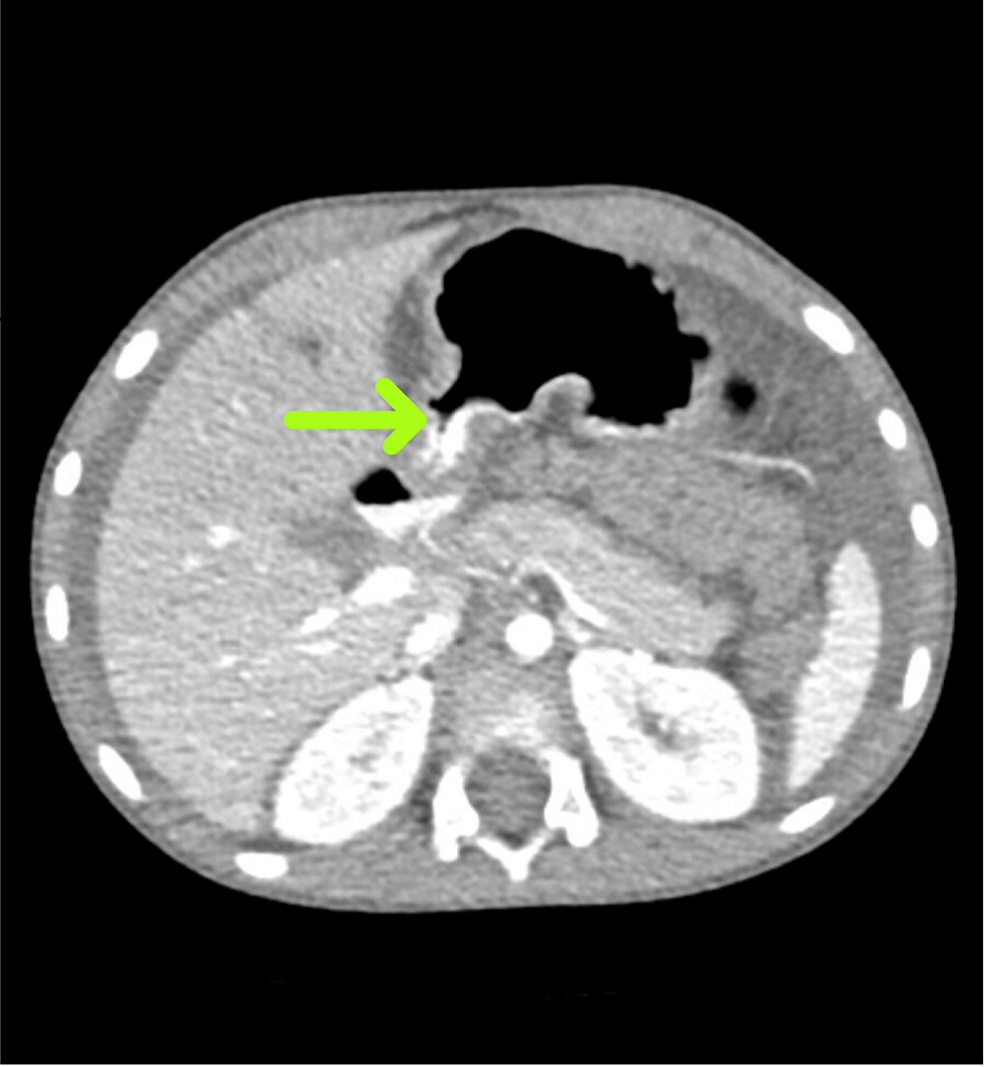
CT scan of the abdomen and pelvis showing possible area where internal hernia was causing obstruction of small bowel loops. Findings were unclear. CT: computed tomography

**Figure 4:**
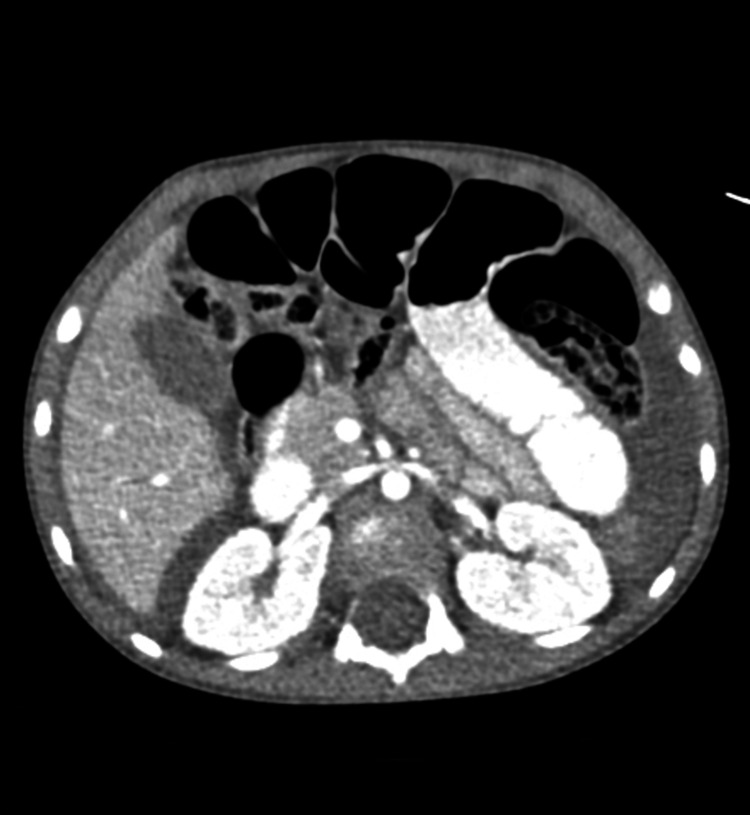
CT scan of the abdomen and pelvis showing no visualization of the appendix, dilated small bowel loops, and surrounding inflammatory changes distal to the area of obstruction. CT: computed tomography

The diagnosis of small bowel obstruction was clear from the patient’s acute presentation and imaging. However, the cause of obstruction remained unclear (Figure 3). Specifically, imaging had ruled out intussusception and the appendix was unable to be visualized (Figure 4). Therefore, the decision was made to perform a stat diagnostic laparoscopy with a possible conversion to laparotomy.

The patient was taken to the operating room where a diagnostic laparoscopy with conversion to laparotomy was performed. There was a 25 cm segment of necrotic ileum identified. Upon further inspection, there was an absence of the mesentery for the distal three-fourths of the small bowel. There was an obvious strangulated internal hernia causing the obstructive ischemia of the aforementioned segment of the bowel. The necrotic section of the bowel was removed and an end-to-end anastomosis was created between the two ends of the bowel. Attention was then turned to the appendix which was subsequently resected and removed. The patient was kept overnight for observation and was discharged with a targeted oral antibiotic course the following day due to an unremarkable post-operative course.

## Discussion

Strangulated internal hernias are a fatal diagnosis unless rapidly diagnosed and operated on. It is important to recognize the signs and symptoms of these hernias to issue prompt treatment. In this case, an ill-appearing two-year-old with a history of vomiting, diffuse abdominal pain, bloating, and guarding upon physical examination warranted a thorough examination and imaging. However, the diagnosis of strangulated transmesenteric hernia was not made until surgery was performed. These hernias typically show nonspecific findings on various abdominal imaging which can be mistaken for other intestinal pathologies such as obstruction, ischemia, or perforation [2]. While the treatment for all of these pathologies requires surgery, it is important to identify an internal hernia as the primary diagnosis to fix the mesenteric defect and prevent a recurrence [3].

Current literature categorizes strangulation as a common occurrence in the case of internal hernias due to mesenteric defects [4]. Other documented cases of strangulated internal hernias in the pediatric population have been presented, diagnosed, and treated in a similar course as the case above. This includes various imaging modalities such as X-ray and CT, surgical resection of the necrotic bowel, and a subsequent antibiotic course. We believe that in suspected cases of strangulated hernias a CT of the abdomen should be ordered prior to any other imaging modality as it has been shown to be the most efficient way of diagnosing an internal hernia by separating it from other causes of intestinal blockage such as adhesions [5]. While other imaging modalities such as ultrasound may offer protection from unnecessary irradiation, this recommendation of administering an abdominal CT in lieu of other imaging modalities, when presented with symptoms consistent with an acute abdomen, comes in light of the fact that an abdominal CT can also serve as an excellent diagnostic tool when evaluating other causes of an acute abdomen such as appendicitis, colitis, and intussusception.

While it appears that most children suffer no adverse outcomes of bowel resection, current literature fails to evaluate the degree of long-term morbidity in these cases. The degree of bowel resection and type of closure should be evaluated further in these populations to establish the most effective treatment plan. Current literature also does not account for screening in newborns with mesenteric defects. While screening and repair of these defects would reduce the number of strangulated hernias, it is unclear if adding this to the current newborn screening will yield significant results.

## Conclusions

Our case highlights the importance of a high index of suspicion with pediatric patients who present with symptoms of acute abdomen with inconclusive imaging results. The knowledge of a definitive criterion for diagnosis and treatment of pediatric strangulated hernias remains to be studied, and a structured protocol for triage of these patients still needs to be widely explored and examined. While strangulated internal hernias as a result of mesenteric defects are the most common, there are various other causes of internal strangulated hernias, both congenital and acquired, that must also be explored with further research. Efforts must also be made to educate parents about having a high index of suspicion, the presentation, and the risks of their children developing a strangulated internal hernia.
